# Maternal Antibody Transfer in Yellow-legged Gulls

**DOI:** 10.3201/eid1507.090036

**Published:** 2009-07

**Authors:** Jessica M.C. Pearce-Duvet, Michel Gauthier-Clerc, Elsa Jourdain, Thierry Boulinier

**Affiliations:** Centre d’Ecologie Fonctionnelle et Evolutive, Montpellier, France (J.M.C. Pearce-Duvet, T. Boulinier); Centre de Recherche de la Tour du Valat, Arles, France (M. Gauthier-Clerc); Kalmar University, Kalmar, Sweden (E. Jourdain)

**Keywords:** Influenza, birds, antibodies, maternally acquired immunity, yellow-legged gulls, viruses, letter

**To the Editor:** Avian influenza viruses (AIVs) are emerging pathogens of concern because they can cause deaths in birds and humans (*1*). Although wild birds likely contribute to AIV emergence because they are the natural reservoir for all known influenza virus subtypes (*1*), our understanding of AIV transmission and immunology in natural avian populations is incomplete (*2*). In this context, the transfer of maternal antibodies is a tool that should be used more often in immunologic analysis. Because antibodies in eggs and hatchlings can reflect the mother’s past exposure to pathogens (*3*,*4*) and both life stages are more easily sampled than adults, quantifying antibodies found in avian young could help clarify AIV epidemiology.

We determined whether eggs of yellow-legged gulls (*Larus michahellis*) contained antibodies against AIVs. Yellow-legged gulls can host AIVs (C. Lebarbenchon, unpub. data), are abundant, and nest in large, dense colonies in coastal areas. In April 2008, we collected 466 eggs from 2 yellow-legged gull colonies located on the Mediterranean coast: 252 eggs from Gruissan (43.1099°N, 3.1071°E; 350 breeding pairs over 1.5 hectares), and 212 from Villeneuve-lès-Maguelone (VLM; 43.4895°N, 3.8520°E; 400 pairs over 1 hectare). Villeneuve nests formed 2 spatially clustered subcolonies: VLM1 (129 eggs) and VLM2 (83 eggs). We also collected global positioning system coordinates for VLM1 nests.

Egg yolks were isolated and frozen at –20°C until analysis. Antibodies were obtained by using chloroform extraction (*4*). The yolk was diluted 1:1 in phosphate-buffered saline and homogenized. An equal volume of chloroform was added, the solution was centrifuged (6,000 × *g* for 15 min), and the supernatant was used in the analyses.

Extracts were tested for antibodies against the AIV nucleoprotein by using a commercial competitive ELISA (IDEXX/Institut Pourquier, Montpellier, France). The assay has been validated by using seagull serum (IDEXX, pers. comm.) and chicken egg yolk (*5*). A subset of samples was tested by using a second commercial competitive ELISA (IDVet, Montpellier, France). Optical density values obtained in the 2 assays were significantly correlated (r = 0.90, df = 39, p<0.001), and serostatus was consistent across assays.

Overall antibody prevalence was 14% (65/466), indicating exposure to influenza A viruses in these colonies. As expected, antibody prevalence in gulls is higher than the viral prevalence previously estimated by reverse transcription–PCR or virus isolation on fecal samples, i.e., the methods typically used by avian influenza surveillance networks. A spring and summer survey performed on feces from gulls of the Camargue region (east of our colonies) showed that only 0.9% of gulls (2 infected of 228 sampled) were excreting AIVs (C. Lebarbenchon, unpub. data).

Egg antibody prevalence did not differ significantly between colonies. The antibody prevalence of 13.5% found at Gruissan (34 of 252 eggs) was comparable with the 14.5% found at Villeneuve (31 of 214 eggs) (generalized linear model with binomial distribution, z = 0.4, p = 0.8). The subcolonies of Villeneuve also did not differ: 18/129 (14%) in VLM1 compared with 12/83 (14.5%) in VLM2 (z = 0.02, p = 0.9). There was no evidence of spatial autocorrelation in the distribution of antibody in eggs by using the Moran I spatial statistic. This similarity of antibody prevalence across and within colonies suggests that exposure is dictated by regional rather than local conditions, a hypothesis that should be tested by sampling across a broader range of nest densities and over time.

Our study presents evidence for the presence of antibodies against AIVs in wild bird eggs, and the findings have important practical implications. The difference in prevalence estimated from virus isolation (0.9%) and antibody detection (14%), although expected, highlights the complementary nature of the 2 approaches. Most surveys estimate current infection by virus isolation, which provides information about disease risk in addition to phylogeographic tracking of strains. In contrast, information on antibody prevalence, which shows past and present population exposure and risk, has largely been ignored with few exceptions (e.g., *6**,**7*). Future work could benefit from using both approaches in tandem with modeling to develop an understanding of avian influenza ecology in nature.

Our results also show the generalizable potential of maternal antibody transfer for tracking pathogen exposure in wild birds, notably in the case of recognized emerging zoonoses. Because eggs and hatchlings are proxies of past and present adult pathogen exposure (*3*,*4*), the difficult and sometimes disruptive sampling of adults can be circumvented by the rapid and cost-efficient sampling of their young, which will facilitate monitoring efforts. Due to the high intranest correlation in egg antibodies (*4*), only partial sampling of clutches (e.g., 1 of 3 eggs) is necessary to track pathogen presence and prevalence through space and time. The sampling effects could further be minimized by taking blood samples from young nestlings of a standardized age. Finally, such samples provide abundant material for the simultaneous surveillance of other emerging pathogens of interest, such as *Campylobacter* spp. (*8*) and West Nile virus (*9*).
